# Supplementary material for: Prevalence, Risk Factors and Course of Osteoporosis in Patients with Crohn’s Disease at a Tertiary Referral Center
Source: J Clin Med. 2019 Dec 10;8(12):2178. doi: 10.3390/jcm8122178 (PMC6947604; doi:10.3390/jcm8122178)
Supplement: Supplementary file 1 [file jcm-08-02178-s001.pdf]

Supplementary:

Table S1: Baseline characteristics of all included patients ≥ 2 DXA scans

**Variable**	***n* = 233**
Male, *n* (%)	103 (44.2)
Age at diagnose of IBD (years), median (range)	23 (8–66)
Age at first DXA scan (years), median (range)	36 (18–77)
Montreal classification of CD:	
Age, *n* (A1:A2:A3)	25:187:21
Location, *n* (L1:L2:L3:L4), *n* = 232	63:35:134:22
Behaviour, *n* (B1:B2:B3), *n* = 231	65:56:110
Disease duration at first DXA scan (years), median (range)	9 (0–42)
Presence of at least one extraintestinal manifestation, *n* (%)	126 (54.1)
Active cigarette smoking at first DXA scan, *n* = 221 (%)	85 (36.5)
BMI (kg/m^2), mean ± SD (range)	23.8 ± 4.8 (14.6–43.1)
History of anti-TNFα treatment, *n* (%)	44 (18.9)
History of anti-integrin treatment, *n* (%)	1 (0.4)
History of anti-interleukin treatment, *n* (%)	0 (0)
History of immunomodulator treatment, *n* (%)	79 (33.9)
History of bowel resection(s), *n* (%)	173 (74.2)
Short bowel syndrome, *n* (%)	6 (2.6)
Ostomy, n (%)	17 (7.3)
BMD, mean ± SD (range)	0.887 ± 0.128 (0.609–1.224)
BMD lumbar spine, mean ± SD (range)	0.939 ± 0.141 (0.623–1.423)
BMD femur, mean ± SD (range)	0.836 ± 0.146 (0.440–1.277)
BMD according to T-score of WHO	
Normal BMD:osteopenia:osteoporosis (*n*:*n*:*n*)	59:112:62
Number of DXA scans per patient	
2, *n* (%)	73 (31.3)
3, *n* (%)	54 (23.2)
4, *n* (%)	39 (16.7)
5, *n* (%)	27 (11.6)
6, *n* (%)	11 (4.7)
7, *n* (%)	8 (3.4)
8, *n* (%)	9 (3.9)
9, *n* (%)	6 (2.6)
10, *n* (%)	2 (0.9)
11, *n* (%)	3 (1.3)
12, *n* (%)	1 (0.4)

BMD: bone mineral density; BMI: body mass index; CD: Crohn's disease; DXA: dual energy X-ray absorptiometry; SD: standard deviation; TNFα: tumor necrosis factor alpha; WHO: world health organization; Montreal classification of Crohn's disease: A1: age < 16 years; A2: age 17–40 years; A3: age > 40 years; L1: location ileal; L2: location colonic; L3: location ileal and colonic; L4: location upper gastrointestinal tract; B1: non-stricturing non penetrating behavior; B2: stricturing behavior; B3: penetrating behavior.

Table S2. Comparison of baseline characteristics between the subgroups of patients with osteoporosis versus those without osteoporosis in patients ≥ 2 DXA scans.

	Osteoporosis	No Osteoporosis	*p*-Value
**Variable**	***n* = 62**	***n* = 171**	
Male, n (%)	34 (54.8)	69 (40.4)	0.049 [1]
Age at diagnosis of CD (years), median (range)	23.5 (8–66)	23 (9–58)	0.210 [2]
Age at first DXA scan (years), median (range)	43 (18–77)	34 (18–67)	0.025 [2]
**Montreal classification of CD:**			
Age			0.001 [1]
A1	7 (11.3)	18 (10.5)	
A2	42 (67.7)	145 (84.8)	
A3	13 (21.0)	8 (4.7)	
Location			0.289 [1]
L1	20 (32.8)	43 (25.1)	
L2	6 (9.8)	29 (17.0)	
L3	35 (57.4)	99 (57.9)	
L4	2 (3.3)	20 (11.7)	0.054 [1]
Behaviour			0.937 [1]
B1	18 (29.0)	47 (27.8)	
B2	14 (22.6)	42 (24.9)	
B3	30 (48.4)	80 (47.3)	
Disease duration at first DXA scan (years), median (range)	10 (0–42)	8 (0–39)	0.290 [2]
Presence of at least one extraintestinal manifestation, *n* (%)	30 (48.4)	96 (56.1)	0.294 [1]
Active cigarette smoking at first DXA scan, *n* (%)	23 (38.3)	62 (38.5)	0.981 [1]
BMI (kg/m^2), mean ± SD (range)	21.2 ± 4.0 (14.6–31.1)	24.7 ± 4.7 (15.2–43.1)	<0.001 [2]
History of anti-TNFα treatment, *n* (%)	12 (18.7)	32 (19.4)	0.912 [1]
History of anti-integrin treatment, *n* (%)	1 (1.6)	0 (0)	0.096 [1]
History of immunomodulator treatment, *n* (%)	22 (35.5)	57 (33.3)	0.759 [1]
History of bowel resection(s), *n* (%)	51 (82.3)	122 (71.3)	0.092 [1]
Short bowel syndrome, *n* (%)	5 (8.1)	1 (0.6)	0.001 [1]
Ostomy, *n* (%)	7 (11.3)	10 (5.8)	0.158 [1]

BMI: body mass index; CD: Crohn's disease; DXA: dual energy X-ray absorptiometry; SD: standard deviation; TNFα: Tumor necrosis factor alpha; [1] Chi-squared test; [2] Mann-Whitney-test; Montreal classification of Crohn's disease: A1: age < 16 years; A2: age 17–40 years; A3: age > 40 years; L1: location ileal; L2: location colonic; L3: location ileal and colonic; L4: location upper gastrointestinal tract; B1: non-stricturing non penetrating behavior; B2: stricturing behavior; B3: penetrating behavior
